# The regulation of sequence specific NF-κB DNA binding and transcription by IKKβ phosphorylation of NF-κB p50 at serine 80

**DOI:** 10.1093/nar/gkz873

**Published:** 2019-10-10

**Authors:** Emma L Smith, Domenico Somma, David Kerrigan, Zoe McIntyre, John J Cole, Kai Ling Liang, Patrick A Kiely, Karen Keeshan, Ruaidhrí J Carmody

**Affiliations:** 1 Centre for Immunobiology, Institute of Infection, Immunity and Inflammation, College of Medicine, Veterinary and Life Sciences, University of Glasgow, Glasgow G12 8TA, UK; 2 Department of Biochemistry, University College Cork, Cork, Ireland; 3 GLAZgo Discovery Centre, Institute of Infection, Immunity & Inflammation, College of Medicine, Veterinary and Life Sciences, University of Glasgow, Glasgow G12 8TA, UK; 4 Department of Life Sciences, and Materials and Surface Science Institute, University of Limerick, Limerick, Ireland; 5 Paul O′Gorman Leukaemia Research Centre, College of Medicine, Veterinary and Life Sciences, Institute of Cancer Sciences, University of Glasgow, Glasgow G12 0ZD, UK

## Abstract

Phosphorylation of the NF-κB transcription factor is an important regulatory mechanism for the control of transcription. Here we identify serine 80 (S80) as a phosphorylation site on the p50 subunit of NF-κB, and IKKβ as a p50 kinase. Transcriptomic analysis of cells expressing a p50 S80A mutant reveals a critical role for S80 in selectively regulating the TNFα inducible expression of a subset of NF-κB target genes including pro-inflammatory cytokines and chemokines. S80 phosphorylation regulates the binding of p50 to NF-κB binding (κB) sites in a sequence specific manner. Specifically, phosphorylation of S80 reduces the binding of p50 at κB sites with an adenine at the −1 position. Our analyses demonstrate that p50 S80 phosphorylation predominantly regulates transcription through the p50:p65 heterodimer, where S80 phosphorylation acts in *trans* to limit the NF-κB mediated transcription of pro-inflammatory genes. The regulation of a functional class of pro-inflammatory genes by the interaction of S80 phosphorylated p50 with a specific κB sequence describes a novel mechanism for the control of cytokine-induced transcriptional responses.

## INTRODUCTION

The transcription factor NF-κB plays an important role in a number of fundamental biological processes including cell cycle, proliferation, differentiation and cell death (1). However, the primary role of NF-κB is as an essential regulator of the immune response through the transcriptional regulation of a large number of inflammatory genes, including chemokines, cytokines and immune effectors (2). The NF-κB transcription factor family is comprised of five structurally related subunits: p65 (RelA), RelB, c-Rel, p50 and p52. The p50 and p52 subunits are generated from the limited proteasomal processing of the precursor proteins p105 and p100 respectively, and lack the transactivation domain (TAD) found in the C terminal regions of the p65, c-Rel and RelB subunits. All NF-κB subunits contain a highly conserved Rel homology domain (RHD) which facilitates dimerisation and DNA binding. NF-κB can promote or repress transcription depending on the subunit composition of dimer complexes. For example, although the p50 subunit lacks a TAD, it can positively regulate transcription by forming a heterodimer with a TAD containing subunit such as p65. Alternatively, p50 homodimers may function as transcriptional repressors by competing with TAD containing NF-κB dimers for the same DNA binding sites in target gene promoters (3).

The primary mechanism regulating NF-κB activity is the cytoplasmic sequestration of NF-κB dimers by the canonical IκB proteins IκB-α, -β and -ϵ, and the p105 and p100 precursor proteins. Activation of NF-κB requires the proteasomal degradation of the IκB proteins triggered by IKK complex (IKKα, IKKβ) mediated phosphorylation of IκBs. The degradation of IκB proteins facilitates the nuclear translocation of NF-κB dimers where they bind to specific κB sites in DNA with the consensus sequence 5′-G^−5^G^−4^G^−3^R^−2^N^−1^W^0^Y^+1^Y^+2^C^+3^C^+4^−3′ (R represents a purine, N represents any nucleic acid, W represents an A or T and Y represents a pyrimidine) (4,5). Although the nuclear localisation of NF-κB is controlled by IκB protein degradation, NF-κB transcriptional activity is regulated by a number of post-translational modifications, including acetylation (6), ubiquitination (7) and phosphorylation (8). The importance of phosphorylation in regulating NF-κB transcriptional activity has been revealed mainly by studies of the p65 subunit, where phosphorylation has been demonstrated to regulate transcription in a stimulus and gene specific manner through a variety of mechanisms including the modulation of p65 interaction with IκBα and other transcription factors, and regulating p65 ubiquitination and stability (8).

Although the NF-κB p50 subunit is a critical regulator of inflammatory gene expression, its regulation by phosphorylation is much less well understood. p50 is one of the most highly expressed transcription factors in macrophages, and is central to macrophage mediated inflammatory responses (9). p50 homodimers are important repressors of inflammatory gene expression and the stability of p50 homodimers is crucial for limiting pro-inflammatory gene expression and establishing Toll-like Receptor tolerance in macrophages (3,10). The phosphorylation of p50 at serine 337 (S337) is required for DNA binding (11), while the phosphorylation of S242 inhibits p50 homodimer DNA binding (12) .Phosphorylation of S20 promotes DNA binding, and is required for VCAM-1 expression in response to TNFα (13). p50 phosphorylation at S328 occurs in response to DNA damage and regulates the interaction of p50 with specific NF-κB binding sites to inhibit anti-apoptotic gene expression (14,15). These studies indicate that transcriptional responses to specific stimuli may be shaped by the integration of signal induced NF-κB phosphorylation and binding site sequence in the regulatory elements of target genes.

In this study, we describe serine 80 (S80) as a novel phosphorylation site on the NF-κB p50 subunit. We identify IKKβ as a S80 kinase and establish p50 as a novel substrate for this kinase. Our data reveals that TNFα-induced phosphorylation of S80 selectively regulates distinct subsets of NF-κB target genes, driven by differential binding of p50 and p65 at specific DNA sequences. Our analyses demonstrate that p50 S80 phosphorylation predominantly regulates transcription through p50:p65 heterodimers and shows that p50 phosphorylation may function in *trans* to inhibit gene transcription. Our analysis demonstrates that S80 phosphorylation reduces the affinity of p50 for κB sites that have an adenine at the −1 position, limiting the expression of genes regulated by these binding sites. Remarkably, the promoters of inflammatory cytokines and chemokines are enriched in κB sites containing a −1 A and are thereby selectively regulated by phosphorylation of p50 at S80. Our data establishes IKKβ phosphorylation of p50 S80 as a novel mechanism that shapes the TNFα-induced transcriptional program and demonstrates the control of gene expression through the interaction of NF-κB phosphorylation and DNA binding site sequence.

## MATERIALS AND METHODS

### Cell culture and transfection

Human embryonic kidney 293T (HEK293T) cells were cultured in DMEM (Sigma), supplemented with 10% fetal bovine serum, 2 mM l-glutamine, and 100 units/ml of streptomycin and penicillin. *Nfkb1*^−/−^ 3T3 MEFs stably expressing p105-XP were generated as previously described (16). Cells were maintained at 37°C in a humidified environment with 5% CO_2_ and sub-cultured by enzymatic detachment with 0.05% Trypsin–EDTA solution (Invitrogen). HEK293T cells were transiently transfected with Turbofect (ThermoFisher Scientific) according to manufacturer's instructions. pFLAG-CMV2 IKKβ, pFLAG-CMV2 IKKβ^K44M^ and pcDNA3 IKKβ^SSEE^ FLAG were kind gifts from Michael May, University of Pennsylvania. pEF4A-p50-XP and pEF4A-p50-Myc were generated by cloning murine cDNA into pEF4A empty vector. pEF4A-p50^S80A^-XP was generated by site directed mutagenesis using the Stratagene QuikChange II SDM kit according to the manufacturer's instructions (Primers: 5′-CCTCTAGTGAGAAGAACAAGAAAGCCTACCCACAGG-3′, 3′-GGAGATCACTCTTCTTGTTCTTTCGGATGGGTGTCC-5′). For generation of CRISPR/Cas9 *NFKB1*^S80A^ knock-in cells, HEK293Ts were transfected using the Neon Transfection System 100 μl kit (ThermoFisher Scientific) using 2 pulses at 1100 V for 20 ms.

### Western blot analysis and immunoprecipitation

Whole cell lysates were generated from cells suspended in radio-immunoprecipitation assay buffer (RIPA) containing 50 mM Tris–HCl (pH 7.4), 1% Nonidet P-40, 0.25% deoxycholate, 150 mM NaCl, 1 mM EDTA, 1 mM PMSF, 1 mM NaF, 1 mM Na_3_VO_4_, 2 μg/ml aprotinin, 1 μg/ml pepstatin and 1 μg/ml leupeptin. Nuclear and cytoplasmic extracts were obtained using a nuclear extraction kit according to the manufacturer's instructions (Active Motif). Protein concentration of lysates were determined using a Bradford assay (Bio-Rad). Immunoprecipitation (IP) assays employed equal concentrations of whole cell extracts, pre-cleared for 30 min at 4°C with protein G-agarose beads (Millipore), and immunoprecipitated with antibody overnight at 4°C. Beads were washed three times in RIPA buffer, resuspended in equal volumes of 2× SDS loading dye and heated for 5 min at 95°C to elute immunoprecipitated protein. Denatured samples were resolved by SDS-PAGE, transferred to nitrocellulose membranes and blocked for 1 h at room temperature using 5% fat free milk powder in PBS–Tween 20. Membranes were immunoblotted with specific antibodies: p-IκBα (cat. # 9246), IκBα (cat. # 4821) and p105/p50 (cat. # 12540) were purchased from Cell Signalling Technologies; antibodies against FLAG (cat. # F1804), HDAC-1 (cat. # AV38530), α-tubulin (cat. # T6074) and β-actin (cat. # A5441) were purchased from Sigma; anti-p65 (cat. # A301-824A) was purchased from Bethyl Laboratories, anti-Myc (cat. # SC-40) was purchased from Santa Cruz; and anti-Xpress (cat. # R910-25) was purchased from Invitrogen. A custom anti-p50 p-S80 antibody was designed and purchased from BioGenes GmbH. Antibody binding was visualised using WesternBright HRP ECL substrate (Advansta) and a C-DiGit chemiluminescence scanner (LiCor).

### Kinase assays

For *in vitro* kinase assays utilising an immunoprecipitated kinase source, cells were transfected with plasmid encoding constitutively active or kinase inactive IKKβ and immunoprecipitations performed as described above. Protein-bound beads were used directly in the kinase assay by adding 800 ng of substrate and 10 μl of kinase assay buffer (400 mM HEPES (4-(2-hydroxyethyl)-1-piperazineethanesulfonic acid) pH 7.5, 400 mM MgCl_2_, 20 mM EDTA, 40 mM NaF, 40 mM β-glycerophosphate, 20 mM DTT, 200 μM cold ATP). 10 μCi γ-32P labelled ATP was added to each sample and incubated at 30°C for 15 min. Samples were made up to 500 μl with ice-cold PBS, incubated with glutathione beads for 30 min and agitated every 5 min. Beads were washed three times with PBS followed by heating at 95°C for 5 min in 40 μl 2× SDS loading buffer. Samples were resolved by SDS-PAGE, and the gel was stained with GelCode Blue (Expedeon) reagent. The gel was fixed in fixation buffer (10% Glacial acetic acid/ 20% methanol/ 50% dH_2_O) for 30 min followed by a 5 min incubation with rehydration buffer (20% methanol/3% glycerol). The gel was dried onto Whatman paper using a gel-dryer, exposed to a phosphor imaging screen overnight and then visualised using the Storm phosphor-imager system (Molecular devices). For recombinant *in vitro* kinase assays, 400 ng recombinant IKKβ kinase (Cell signalling technologies, #7458) was incubated with 400 ng substrate following the protocol as described above. For the peptide array kinase assay, peptide libraries of murine p50 were generated by an automatic SPOT synthesis, as previously described (17) and synthesised on continuous cellulose membrane supports on Whatman 50 cellulose using Fmoc (*N*-(9-flurenyl)methoxycarbonyl) chemistry with AutoSpot-Robot ASS 222 (Intavis Bioanalytical Instruments). The array was used as a substrate for immunoprecipitated and recombinant IKKβ, carried out as described by (18).

### Site directed mutagenesis and GST protein purification

p50 was cloned into pGEX-6P1 in frame with the N-terminal GST tag and transformed into BL21 CodonPlus *Escherichia coli* (Stratagene). Transformants were grown to an *A*_600_ of 1.0–2.0 at 37°C and induced with 1 mM isopropyl β-d-1-thiogalactopyranoside (IPTG) for 16 h at 20°C. The bacteria were resuspended in a buffer containing 50 mM Tris (pH 8.0), 150 mM NaCl and 1 mM dithiothreitol, disrupted by sonication, and centrifuged to remove debris. Recombinant proteins were affinity-purified with GSH-agarose (Sigma) and eluted with 10 mM glutathione (Promega) in 50 mM Tris (pH 8.5) and 150 mM NaCl. GST-p50 mutants were created by PCR based site-directed mutagenesis using the Stratagene QuikChange II SDM kit, according to manufacturer's instructions and recombinant protein purified as above.

### CRISPR/Cas9 genome editing

pSpCas9(BB)-2A-GFP (PX458) plasmid was a gift from Feng Zhang (Addgene plasmid # 48138). gRNA oligonucleotides were purchased from Eurofins Genomics (5′-CACC GACAAACTTACTTTGACCTGA-3′, 3′-CTGTTTGAATGAAACTGGACTCAAA-5′) and were cloned into pSpCas9(BB)-2A-GFP. ssODN was purchased from Integrated DNA Technologies (5′-AGAGGATTTCGTTTCCGTTATGTATGTGAAGGCCCATCCCATGGTGGACTACCTGGTGCCTCTAGTGAAAAGAACAAGAAAGCTTATCCTCAGGTCAAAGTAAGTTTGTGGTAGCTCTCCTTCTATTTGAATTCTGGAAATTTTGATTTCCTACGATTTCCAAGGAATT-3′). The ssODN contains synonymous codon changes designed to introduce the S80A mutation, a HindIII restriction site, and to abolish the PAM site. Prior to transfection, HEK293T cells were treated with 200 ng/ml Nocodazole (Sigma) for 17 h. Following treatment, cells were washed twice with media, trypsinised and cell density was determined. Cells were washed with Mg^2+^ and Ca^2+^ free PBS, and re-suspended in Neon buffer R (ThermoFisher Scientific) at a density of 5 × 10^7^ cells/ml per transfection. 100 μl of cells were co-transfected with pPGKpuro (a gift from Rudolf Jaenisch, Addgene plasmid # 11349), pX458 Cas9/sgRNA vector and ssODN (1:2:2 ratio) by electroporation. Transfected cells were plated into a 10 cm dish containing 10 ml media and recovered overnight in culture. Transfected cells were then selected for ∼3 days with 3 μg/ml of puromycin. After selection, puromycin media was removed and cells washed and incubated in fresh media. Serial dilution of selected cells was performed to isolate single cell clones in 96-well plates. For clone screening, genomic DNA was extracted from cells using the DNeasy Blood and Tissue Kit (QIAGEN) according to the manufacturer's instruction. DNA was amplified by PCR using primers (F: 5′-ACCTGGCTTTTTAGCCATATCT-3′; R: 5′-TTCAGCTTAGGAGCGAAGGC-3′) and HotStarTaq Master Mix Kit (QIAGEN) according to the manufacturer's instructions. Initial screens were performed by HindIII (NEB) restriction digest of PCR products purified using QIAquick PCR Purification Kit (QIAGEN), according to the manufacturer's instructions. Gene editing of selected clones was confirmed by Sanger sequencing (GATC-Biotech).

### Chromatin immunoprecipitation

HEK293T cells were cultured with or without 10 ng/ml TNFα for 1 h before ChIP assays were performed. ChIP assays were performed using the Pierce Magnetic ChIP Kit (Thermofisher Scientific) according to manufacturer's instructions, with one deviation. Following MNase digestion, nuclei were lysed on ice for 10 min in SDS lysis buffer containing 1% SDS, 50 mM Tris (pH 8.0), 10 mM EDTA, 1 mM PMSF, 1 mM NaF, 1 mM Na_3_VO_4_, 2 μg/ml aprotinin, 1 μg/ml pepstatin and 1 μg/ml leupeptin. Lysed cells were centrifuged to remove debris. DNA-bound protein was immunoprecipitated using anti-p105/p50 antibody (Cell signalling technologies, cat. # 12540), anti-p65 (Bethyl Laboratories, cat. # A301-824A) or rabbit IgG (Cell Signalling Technologies cat. # 2729), as a control. The eluted DNA was quantified by real-time PCR using specific primer sets flanking the expected κB site in each gene: *IL8* F: 5′-GGGCCATCAGTTGCAAATC-3′; R: 5′-GCTTGTGTGCTCTGCTGTCTC-3′;*CXCL1* F: 5′-ACTCGGGATCGATCTGGAACTC-3′; R: 5′-CTCCGAGATCCGCGAACCC-3′; *CXCL2* F: 5′-ATTCGGGGCAGAAAGAGAAC-3′; R: 5′- ACCCCTTTTATGCATGGTTG-3′;

### Transcriptomic and bioinformatic analysis

For RNA-sequencing, total RNA was extracted from cells using RNeasy kits (QIAGEN), according to the manufacturer's instructions. Triplicate independently generated samples for each condition were sequenced to a read depth of 20 million using Illumina NextSeq™500 platform. Single-end 75 bp reads were aligned to human reference sequence iGenome NCBI GRCh38 using HISAT (19). Aligned reads were counted using HTseq-count (20). The above analyses were performed using the University of Glasgow Galaxy server. Differentially expressed genes were calculated and visualised using an in-house RNA-seq analysis pipeline which utilises DESeq2 (21). Induced genes with adjusted *P* value <0.05 were included in this study. Heat maps of gene groups were produced using DESeq2 mean normalised counts and visualized using the online tool Morpheus (Broad Institute). For real-time quantitative PCR, total RNA was extracted from cells using RNeasy kits (QIAGEN) according to the manufacturer's instructions and quantified using NanoDrop 1000 Spectrophotometer (ThermoFisher Scientific). 1 μg of isolated RNA was primed with random hexamer oligonucleotides and reverse transcribed using Primer Design precision nanoScript reverse transcription kit. 10 μl PCR reactions were performed with 1 μl cDNA (diluted 1:5), PerfeCTa SYBR Green FastMix (Quantabio) and QIAGEN QuantiTect primers (*BCL3* cat. # QT00008050; *CSF1* cat. # QT00035224; *CXCL2* cat. # QT00013104; *IL8* cat. # QT00000322; *MAP3K8* cat. # QT00051730; *TBP* cat. # QT00000721; *TNF* cat. # QT00029162) using QuantStudio 7 Flex Real-Time PCR System (ThermoFisher Scientific). Thermocycling conditions consisted of 94°C for 20 s, followed by 40 cycles of 95°C for 1 s and 60°C for 20 s. Melt curves for qPCR primers are shown in Supplementary Figure S1. All data were normalised to *TBP*. Gene expression changes were calculated using the 2^−ΔΔCT^ method.

### Motif analysis

Motif analysis was based on the GREAT approach (22), which incorporates both proximal and distal regulatory regions for enrichment analyses. Regulatory regions for genes were obtained from the Ensembl Regulatory Build (23). Genomic locations were obtained using the ‘fetch closest non-overlapping feature’ tool. JASPAR Position Weight Matrix MA0105.1 was used to identify the best matching NFKB1 binding sites in regulatory regions using the ‘FIMO’ tool. These analyses were performed using the University of Glasgow Galaxy server. Sequence logos were generated using WebLogo (24). Transcription factor binding site shape analysis was performed using the TFBSshape online tool (25).

### DNA affinity binding assay (DAPA)

5′-Biotinylated and unlabelled single-stranded oligonucleotides containing a central κB site flanked either side a by 7-bp spacer were purchased from Eurofins Genomics (5′-AGTTGAGGGGNNTTTCCCAGGC-3′; 3′-TCAACTCCCCNNAAAGGGTCCG-5′, where N represents −2 −1 κB site variation per assay). Oligonucleotides were annealed to create 5′-biotinylated, and unlabelled double stranded duplexes. DAPA reactions were prepared by mixing 1.5 μg of 5′-biotinylated double stranded oligo with 150 μg of nuclear extract and 15 μl streptavidin-agarose beads (Sigma) in a total of 500 μl DAPA buffer (10 mM Tris–HCl pH 7.5, 50 mM NaCl, 1 mM DTT, 5% glycerol, 1 mM EDTA, 1 mM NaF, 1 mM Na_3_VO_4_, 1 mM PMSF, 1 μg/ml leupeptin, 2 μg/ml aprotinin, 2 μg/ml pepstatin). Unlabelled double stranded oligonucleotides were added in 10-fold excess to confirm binding specificity. Reactions were incubated at room temperature on a rotator for 1 h. Beads were washed three times in 500 μl DAPA buffer. To elute DNA-bound proteins, beads were resuspended in 20 μl of 2× SDS sample buffer, and incubated at 95°C for 5 min. Eluates were resolved on SDS-PAGE gels and analysed by western blot.

### Luciferase assays

Promoters containing four sequential copies of defined κB sites (5′-GGGAATTTCC-3′, 5′-GGGACTTTCC-3′, 5′-GGGGATTTCC-3′, 5′-GGGGCTTTCC-3′) were cloned into the pTAL-Luc vector (Clontech). WT and *NFKB1*^S80A^ HEK293T cells were co-transfected with 100 ng pTAL-(4xκB) Luc vector and 10 ng *Renilla* luciferase expression vector pRL-TK (Promega). Twenty four hours post transfection, cells were cultured with or without 10 ng/ml TNFα for 8 h before harvest. Luciferase activities of whole cell lysates were analysed using the Dual-Luciferase Reporter Assay System (Promega). The ratio of firefly to *Renilla* luciferase activity was used to normalise for transfection efficiency across all samples.

### Limited proteolysis

Whole cell lysates were generated from cells suspended in RIPA buffer lacking protease inhibitors. Limited proteolysis was performed by adding varying ratios of trypsin (Sigma) to 50 μg of whole cell lysate, and incubating the reaction mixture for 30 min at 37°C. Proteolysis was terminated by adding 5× SDS sample buffer to the reaction and heating at 95°C for 5 min. Samples were resolved by SDS-PAGE and analysed by western blot analysis.

## RESULTS

### IKKβ phosphorylates NF-κB p50

**Figure 1.**
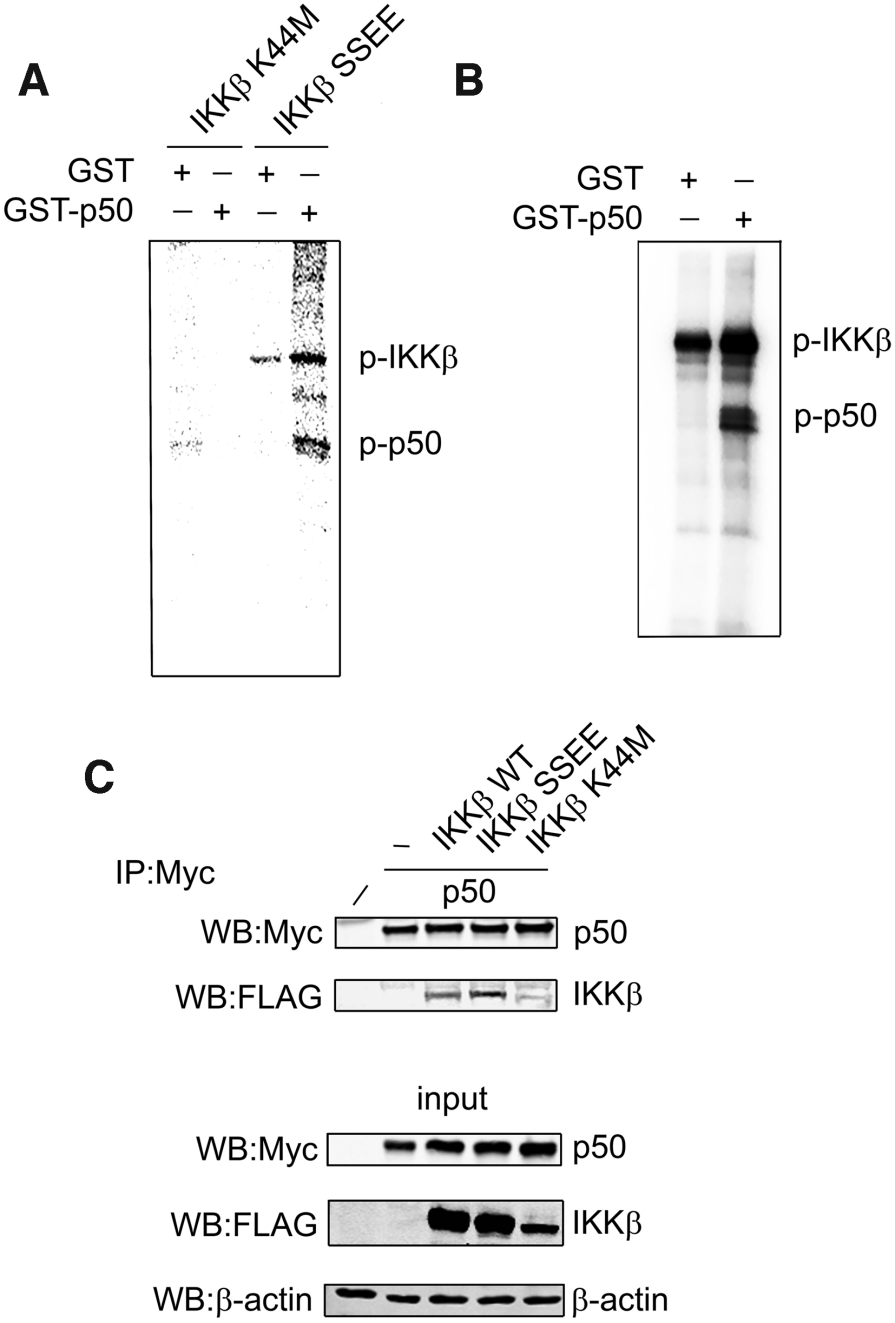
IKKβ phosphorylates NF-κB p50. (**A**) HEK293T cells were transfected with FLAG-IKKβ^K44M^ (kinase dead) or FLAG-IKKβ^SSEE^ (constitutively active) as indicated. IKKβ was immunoprecipitated from whole cell lysates with anti-FLAG antibody and incorporated in a kinase assay using recombinant GST-p50 or recombinant GST. (**B**) *In vitro* kinase assay employing recombinant IKKβ and recombinant GST-p50 or GST. (**C**) HEK293T cells were co-transfected with Myc-p50 and FLAG-IKKβ^SSEE^ or FLAG-IKKβ^K44M^ as indicated. p50 was immunoprecipitated (IP) from whole cell lysates with anti-Myc antibody and analysed by western blot (WB) using the indicated antibodies.

The phosphorylation of NF-κB subunits is strongly linked to the activation of the NF-κB pathway. Indeed, in addition to IκBα, IKKβ also phosphorylates other components of the NF-κB pathway including p65 and p105 (8). To determine if IKKβ may also phosphorylate p50, we initially performed *in vitro* kinase assays using constitutively active IKKβ (IKKβ^SSEE^) immunoprecipitated from transiently transfected HEK293T cells and employing purified recombinant GST-p50 as substrate. These assays revealed that active IKKβ phosphorylates p50 *in vitro* (Figure 1A). Kinase assays incorporating immunoprecipitated kinase dead IKKβ (IKKβ^K44M^) and purified recombinant GST established that p50 phosphorylation was due to IKKβ kinase activity (Figure 1A). Similar results demonstrating the phosphorylation of p50 by IKKβ were obtained using *in vitro* kinase assays incorporating purified recombinant IKKβ (Figure 1B). Co-immunoprecipitation of p50 and constitutively active IKKβ^SSEE^ in transfected cells demonstrated that IKKβ and p50 interact (Figure 1C) as previously reported (26–29). Of note, p50 interacted with the active but not a kinase dead form of IKKβ indicating that IKKβ activity is required for interaction with p50 (Figure 1C). Specificity of co-immunoprecipitation of p50 with IKKβ was validated using an irrelevant IgG under the same conditions (Supplementary Figure S2). These data establish the p50 subunit of NF-κB as a novel substrate of the IKKβ kinase.

### Serine 80 is the IKKβ phosphorylation site of NF-κB p50

**Figure 2.**
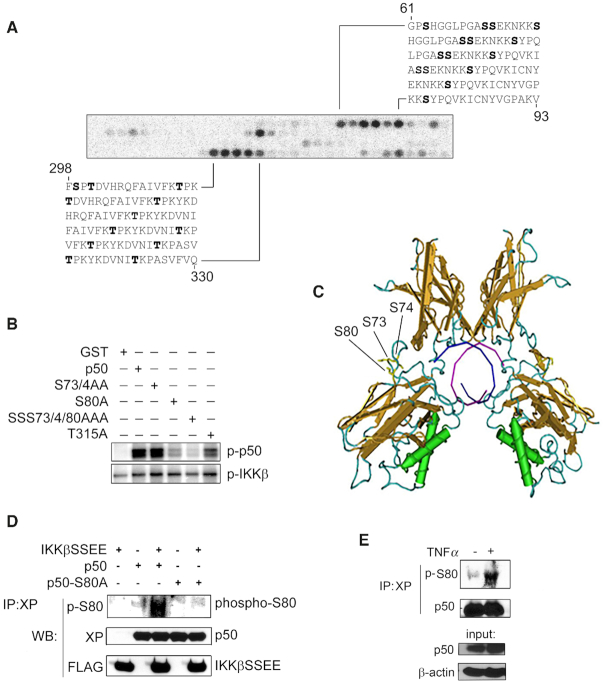
IKKβ phosphorylates NF-κB p50 at S80. (**A**) *In vitro* IKKβ kinase assay using a peptide array of immobilised, overlapping 18-mer peptides representing the entire p50 sequence. Black spots represent IKKβ-phosphorylated peptides. Peptide sequences, amino acid number (mouse p50) and putative phosphosites (bold) are shown. (**B**) *In vitro* IKKβ kinase assay using recombinant GST, wild type and mutated recombinant GST p50 as indicated. (**C**) Image from the X ray crystal structure of p50 homodimer bound to DNA (1NFK) indicating the location of S80, S73 and S74 in an extended loop of the Rel Homology Domain. T315 is not represented in this crystal structure. (**D**) HEK293T cells were co-transfected with or without FLAG-IKKβ^SSEE^ and with either XP-p50 or XP-p50^S80A^ as indicated. p50 was immunoprecipitated (IP) from whole cell lysates with anti-XP antibody and analysed by western blot (WB) using anti-phospho-serine 80 (p-S80) antibody. (**E**) *Nfkb1*^*-/-*^3T3 MEFS stably expressing XP-tagged p105 were stimulated with TNFα (10 ng/ml) for 15 min and p50 immunoprecipitated using anti-XP antibody. Immunoprecipitates were analyse by western blot using anti-phospho-S80 antibody as indicated.

To identify the specific amino acids of p50 phosphorylated by IKKβ we employed an *in vitro* kinase assay using a peptide array representing the entire amino acid sequence of p50. A series of 30, 18-amino-acid-long peptides were SPOT synthesised on nitrocellulose, with each peptide overlapping by three residues to generate a p50 peptide array. The p50 array was subjected to *in vitro* kinase assays using recombinant IKKβ as previously described (18). This analysis identified four putative IKKβ phosphorylation sites in p50; S73, S74, S80 and T315 (human p50 amino acid numbering used) (Figure 2A). To further test the putative sites of IKKβ phosphorylation we next performed *in vitro* kinase assays incorporating recombinant IKKβ and recombinant GST-p50 in which S73, S74, S80 and T315 are mutated to alanine. This analysis revealed that IKKβ could still phosphorylate p50 when S73, S74, and T315 are mutated to alanine, but phosphorylation of p50 is significantly reduced when S80 is mutated to alanine (Figure 2B). These data identify S80 of p50 as the major site of IKKβ phosphorylation. Analysis of the available crystal structure of p50 homodimer bound to DNA revealed that S80 of p50 is located in an extended loop of the RHD and therefore available for phosphorylation (Figure 2C). To further confirm IKKβ phosphorylation of p50 *in vivo*, we next generated an antibody raised against the S80 phosphorylation site of p50. This anti-phospho-S80 p50 antibody recognised IKKβ dependent phosphorylation of p50 but not a p50^S80A^ mutant in transiently transfected cells, demonstrating the phosphorylation of p50 S80 by IKKβ in cells (Figure 2D). To assess the inducible phosphorylation of S80 we stimulated *Nfkb1^−^^/-^* 3T3 MEFs stably expressing XP-tagged p105 (16) with TNF for 15 mins prior to immunoprecipitation with anti-XP antibody and immunoblot analysis using anti-phosphor S80 antibody. This revealed TNFα induced phosphorylation of S80 after 15 min, consistent with the rapid activation of IKKβ by TNFα (Figure 2E). Unfortunately, this antibody was not of sufficient affinity to generate a specific signal in cell lysates of non-transfected cells and so was not of further use in investigating p50 S80 phosphorylation.

### S80 phosphorylation is not required for NF-κB activation

**Figure 3.**
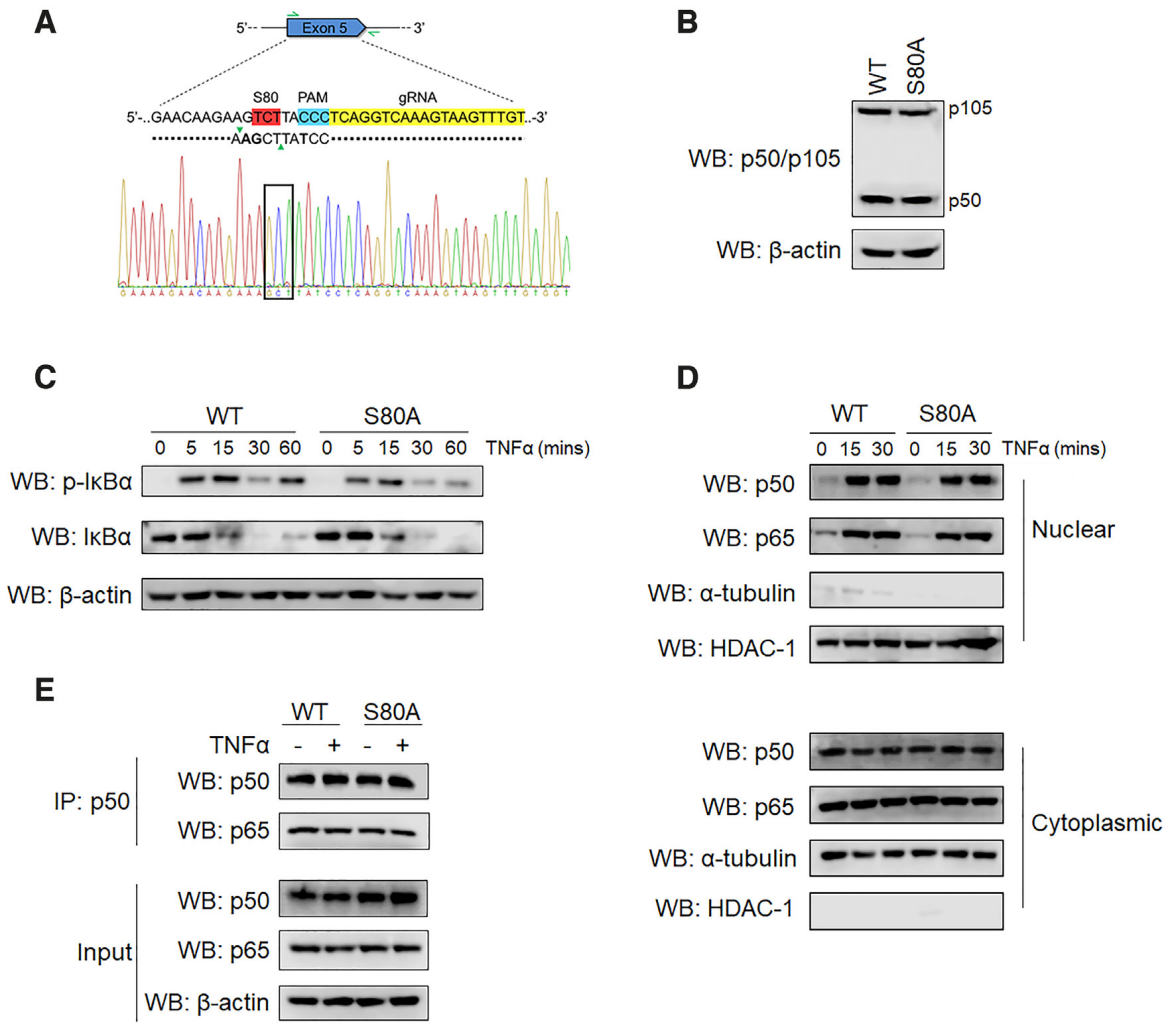
S80 phosphorylation is not required for NF-κB activation. (**A**) Schematic of the CRISPR/Cas9 targeting strategy used to edit the *NFKB1* target locus, highlighting the position of S80 (red); gRNA (yellow); PAM site (blue) and primers used for screening (green arrows). The ssODN template including homology arms (dotted line), HindIII restriction cut site (green triangles) and synonymous codon changes (bold) are shown. Also shown is DNA sequence chromatogram of the *NFKB1* target region confirming the S80A point mutation (black box). (**B**) Whole cell lysates extracted from WT and *NFKB1*^S80A^ HEK293Ts were analysed by western blot (WB) with the indicated antibodies. (**C**) WT and *NFKB1*^S80A^ HEK293Ts were stimulated with 10 ng/ml TNFα for the indicated times prior to lysis. Whole cell extracts were analysed by western blot to detect levels of phosphorylated and total IκBα protein. (**D**) WT and *NFKB1*^S80A^ HEK293Ts were stimulated with 10 ng/ml TNFα for the indicated times prior to lysis. Nuclear and cytoplasmic extracts were analysed by western blot using antibodies against p65 and p105/p50. (**E**) WT or *NFKB1*^S80A^ HEK293Ts were left untreated or were stimulated with 10 ng/ml TNFα for 30 min prior to lysis. p50 was immunoprecipitated (IP) from whole cell lysates with anti-p105/p50 antibody and analysed by western blot (WB) with anti-p105/p50 and anti-p65 antibodies as indicated.

Following the identification of S80 as a novel phosphorylation site on the p50 subunit, we next sought to investigate its role in regulating NF-κB activity. To achieve this we utilised CRISPR/Cas9 genome editing techniques to generate *NFKB1*^S80A^ knock-in HEK293T cells (Figure 3A). The p50 subunit of NF-κB is generated from the proteasomal processing of the p105 precursor which requires the IKKβ mediated phosphorylation at the C terminus of p105 (28–31). To determine whether S80 plays a role in the processing of p105 we analysed p105/p50 protein levels in whole cell lysates from wild type (WT) and *NFKB1*^S80A^ cells by western blot. This revealed equivalent levels of p105/p50 in WT and *NFKB1*^S80A^ cells demonstrating that S80 is not required for the processing of p105 to p50 (Figure 3B). To determine the effect of S80 mutation on the activation of the NF-κB pathway we next stimulated WT and *NFKB1*^S80A^ cells with TNFα and assessed the phosphorylation and degradation of IκBα by western blot. This analysis demonstrated equivalent levels of IκBα phosphorylation and degradation in WT and *NFKB1*^S80A^ cells stimulated with TNFα (Figure 3C). Nuclear translocation of p50 and p65 in TNFα stimulated WT and *NFKB1*^S80A^ cells was assessed by immunoblot analysis of nuclear and cytoplasmic fractions. This demonstrated equivalent levels of NF-κB translocation to the nucleus following TNFα stimulation (Figure 3D). Together, these data demonstrate that upstream signalling and nuclear translocation of NF-κB following TNFα stimulation is unaffected by the p50 S80A mutation. Furthermore, no significant differences in proliferation or cell death were observed between WT and *NFKB1^S80A^* cells. To investigate the effect of S80 mutation on p50 interaction with p65 we performed an immunoprecipitation assay using anti-p50 antibody. Western blot analysis of immunoprecipitates showed that equivalent levels of p65 co-purified with p50 in both WT and *NFKB1*^S80A^ cells demonstrating that p50 S80A mutation does not alter the interaction of p50 and p65 (Figure 3E).

### S80 phosphorylation selectively regulates TNFα-induced gene expression

**Figure 4.**
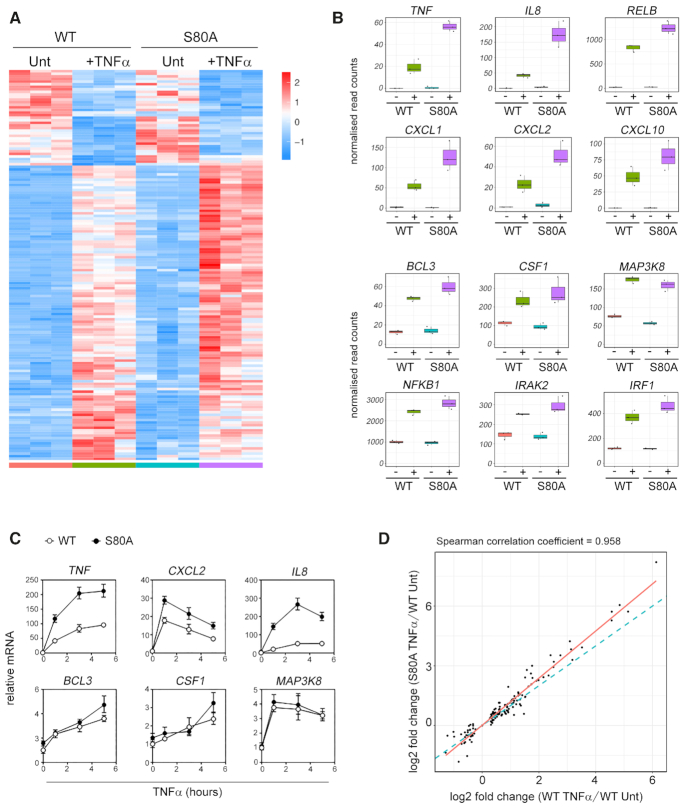
S80 phosphorylation selectively regulates TNFα-induced gene expression. (**A**) Triplicate samples of WT and *NFKB1^S80A^*cells treated with TNFα for 3 h (+TNFα) or untreated (Unt) were analysed by RNA-seq. The heat map displays differentially expressed genes (*P*_adj_ <0.05) scaled as per *z*-score. Genes were clustered using spearman distances and UPGMA agglomeration. (**B**) Box and whisker plots of gene expression level by RNA-seq for selected genes. Each dot represents a sample. Boxes show the 25th, 50th and 75 percentile with whiskers showing inter-quartile range. Untreated = -; TNFα treated = +.(**C**) WT and *NFKB1*^S80A^ HEK293Ts were stimulated with 10 ng/ml TNFα for the indicated times prior to harvest and RNA extraction. Gene expression levels were determined by quantitative real-time PCR. Data are mean ± S.E of triplicate samples and are representative of three independent experiments. (**D**) Log_2_ fold change scatter plot of genes (dots) shown in (B). The blue dotted line indicates correlation the between WT TNFα/WT Unt v S80A 0hr/WT Unt fold changes (spearman correlation ∼0.96). The red line represents linear regression. Data are mean of three independent experiments.

Site-specific phosphorylation of NF-κB subunits has previously been shown to regulate transcriptional activity (8). To determine the role of S80 phosphorylation in regulating NF-κB target gene expression, we next analysed TNFα-induced transcriptional responses in WT and *NFKB1^S80A^* cells by RNA-seq. WT and *NFKB1^S80A^*cells were untreated or treated with TNFα for 3 h prior to RNA-seq analysis. This revealed distinct transcriptional profiles of differentially expressed genes in WT and *NFKB1^S80A^* cells in response to TNFα (Figure 4A, Supplementary Figure 3 and Supplementary Table S1). In particular we identified two TNFα-inducible groups of genes composed predominantly of NF-κB target genes containing an identifiable NF-κB binding site in their promoter regions that were differentially regulated between WT and *NFKB1^S80A^* cells (Figure 4B). The expression of genes encoding pro-inflammatory chemokines and cytokines including *TNF*, *IL8*, *CXCL2*, *CXCL1* and *CXCL10* was dramatically increased in *NFKB1^S80A^* cells compared to WT cell following TNFα treatment. However, other NF-κB target genes predominantly encoding for intracellular signalling factors including *BCL3*, *MAP3K8*, *NFKB1* and *IRAK1* were expressed at equivalent levels in both WT and *NFKB1*^S80A^ cells following TNFα treatment. Analysis of selected genes from each group by qPCR confirmed the gene-selective regulation of transcription by p50 S80 phosphorylation (Figure 4C). Of note, although the expression levels of a number of TNFα-inducible genes is higher in *NFKB1^S80A^* cells than WT cells, the gene set induced by WT and *NFKB1*^*S80A*^ cells is highly similar (Figure 4D). This indicates that S80 phosphorylation regulates the levels of gene expression induced by TNFα treatment rather than the specific genes induced.

### Specific DNA-binding motifs associated with differential regulation of NF-κB target genes by S80

**Figure 5.**
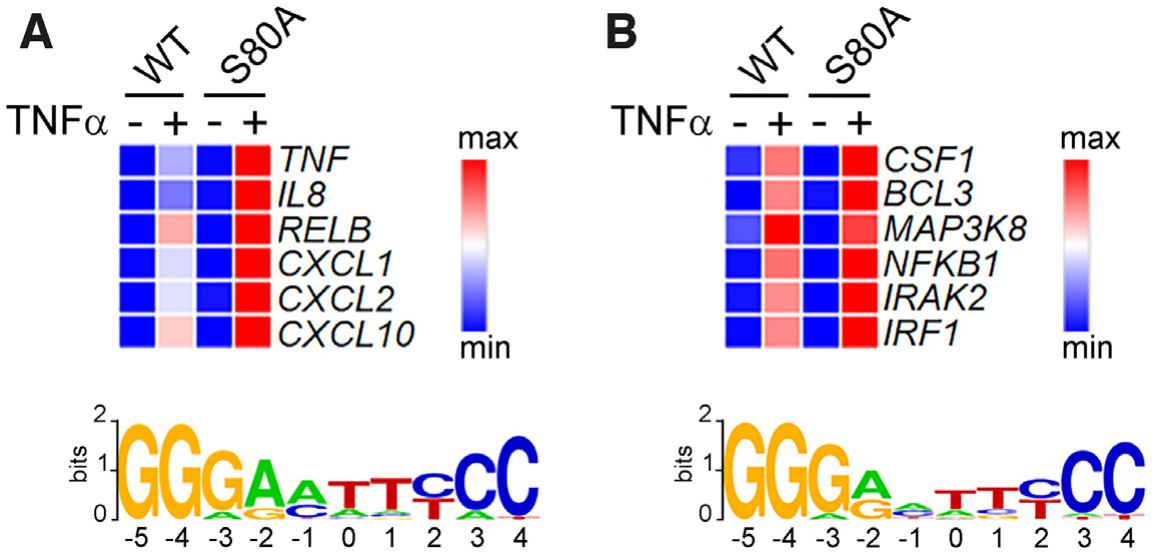
Specific κB sites are enriched in genes selectively regulated by S80 phosphorylation. Gene expression values (scaled as per *z*-score) of selected genes (**A**) induced to a greater degree in NFKB1^S80A^ cells compared to WT cells, and (**B**) genes expressed equally between WT and NFĸB1^S80A^ cells. Proximal and distal regulatory regions surrounding each gene were searched for occurrences of *NFKB1* motifs best matched to JASPAR position weight matrix MA0105.1. Sequence logos representing the most enriched κB site for each gene set are shown.

The gene selective effects of S80 mutation on TNFα-induced transcription suggested that phosphorylation of S80 may regulate the activity of p50 in a promoter specific manner. Binding site sequence preferences differ among NF-κB dimers (32) and suggests a potential mechanism for the selective effect of S80 mutation on the transcription of a distinct set of target genes. To further explore this possibility, we performed transcription factor binding site analysis to search for the most enriched κB sites in TNFα-induced genes expressed higher in *NFKB1^S80A^* cells than WT cells, and those expressed equally in both WT and *NFKB1^S80A^* cells. The genomic region between the nearest upstream and downstream gene was used to search for both proximal and distal regulatory features (including promoters and enhancers) for each gene. Regulatory features were analysed for occurrences of a 10 base pair *NFKB1* position weight matrix (JASPAR ID MA0105.1). This analysis identified distinct DNA binding motifs associated with each group of genes (Figure 5A and B). Specifically, promoter and enhancer κB sites varied at the −1 and −2 positions (Figure 5A and B). The κB sites of genes with increased expression in *NFKB1*^S80A^ cells relative to WT cells, contain an adenine (A) at the −2 position, and either an adenine or a cytosine (C) at the −1 position (Figure 5A). In contrast, the κB sites of genes where expression is unchanged between WT and *NFKB1^S80A^* cells contain either an A or a guanine (G) at the −2 position while there is no enrichment of any particular nucleotide at the −1 position. This analysis suggested that S80 phosphorylation may regulate p50 function in a DNA binding site sequence specific manner.

### S80 phosphorylation reduces p50 affinity for −1A containing κB sites

**Figure 6.**
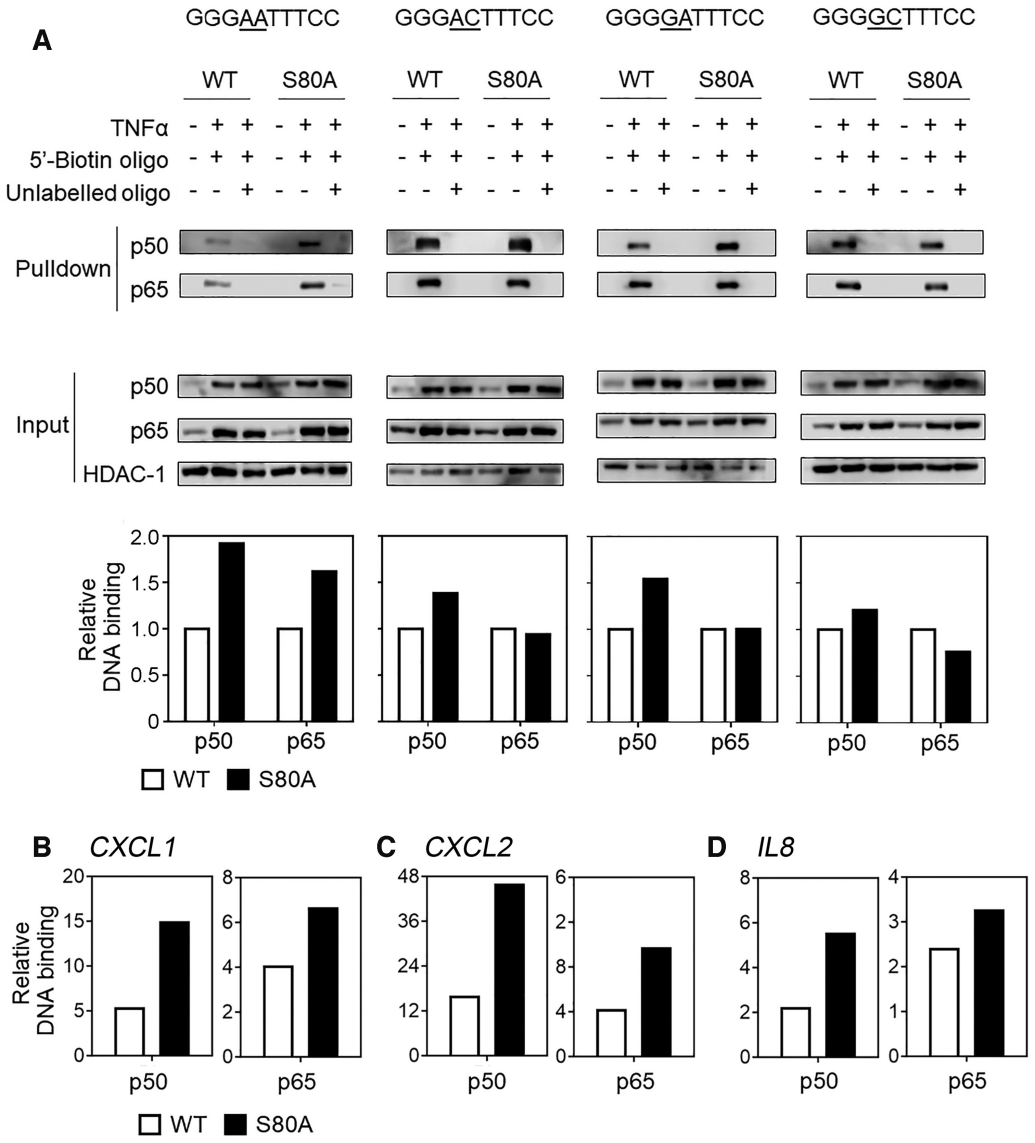
S80 phosphorylation regulates DNA binding affinity to −1A containing κB sites. (**A**) WT and *NFKB1*^S80A^ HEK293T were left untreated or treated with 10 ng/ml TNFα as indicated for 30 min. Equal concentrations of nuclear lysates were incubated with 5′-biotinylated double stranded oligonucleotides, with or without a 10× excess of unlabelled competitor double stranded oligo as indicated. The sequence of the double stranded oligos containing κB-sites that vary at the −1 and −2 positions are indicated for each assay (underlined). Precipitated samples were analysed for p50 and p65 proteins by WB. Relative binding of p50 and p65 protein to DNA was quantified by normalising to input. Data shown are representative of three individual experiments. (**B**–**D**) Chromatin immunoprecipitation of p50 and p65 from TNFα stimulated WT and *NFKB1^S80A^* cells. Primers flanking the NF-κB binding sites of the *CXCL1*, *CXCL2* and *IL8* promoters were used to assess DNA binding. Data is representative of 3 independent experiments.

To determine if S80 phosphorylation alters the binding of p50 to specific κB sequences we next carried out DNA affinity precipitation assays (DAPA) incorporating the κB sequences identified in our analysis of transcriptomic data (Figure 5). WT or *NFKB1*^S80A^ cells were left untreated or treated with TNFα prior to extraction of nuclear fractions. Nuclear extracts were incubated with 5′-biotinylated oligonucleotides representing κB sites with base pair substitutions at the −1 and −2 positions. The oligonucleotides were designed to represent the DNA-binding motifs identified in the gene sets identified by our transcriptomic analysis (Figure 5A and B), and differed only in the base pair sequence at the −2 and −1 positions of a GGGA(−2)C(−1)TTTCC motif. Oligonucleotides representing κB sites from genes with enhanced TNFα inducible expression in *NFKB1^S80A^* cells therefore contained an A at the −2 position and an A or C at the −1 position. Since the κB sites identified in genes with equivalent expression in both WT and *NFKB1^S80A^* cells displayed heterogeneity at the −1 position we generated oligonucleotides with an A or C at the −1 position to reflect the previously reported prevalence of A and C at this position of κB sites (33) and a G at the −2 position. Oligonucleotides and bound protein were precipitated using streptavidin conjugated agarose beads. Specificity of protein-DNA interaction was verified using control samples containing a tenfold excess of non-biotinylated oligonucleotide. Precipitated proteins were resolved by SDS gel electrophoresis and analysed by western blot using antibodies against p50 and p65 and quantified using a digital chemiluminescence scanner. These assays demonstrated approximately 2 fold greater binding of p50^S80A^ to the GGGA(−2)A(−1)TTTCC κB site when compared to WT p50 (Figure 6A). Remarkably, there is also significantly greater binding of p65 to this κB site in *NFKB1^S80A^* cells relative to WT cells, demonstrating that p50 S80 phosphorylation regulates the DNA binding of p50:p65 heterodimers. The requirement of an A at the −1 position for this effect was demonstrated by a single base change of −1A to −1C in the GGGA(−2)C(−1)TTTCC ĸB site which largely abolished the increased p50^S80A^ binding and completely abolished the increased p65 binding seen with the GGGA(−2)A(−1)TTTCC site (Figure 6A).

The significance of the −1A nucleotide to p50^S80A^ binding was further demonstrated by the increased binding of p50^S80A^ to a GGGG(−2)A(−1)TTTCC ĸB sites relative to WT p50 (Figure 6A). Of note, the levels of p65 binding to this site were similar in both p50^S80A^ and WT p50 expressing cells indicating that S80 phosphorylation may regulate the binding of p50:p50 homodimers but not p50:p65 heterodimers to κB sites with this sequence. This suggested that binding to specific κB sequences may also be influenced by the composition of p50 containing dimers. An additional change from A to C in the −1 nucleotide position in the GGGG(−2)C(1)TTTCC site further reduces p50^S80A^ binding relative to WT p50 as compared to GGGG(−2)A(−1)TTTCC κB sites (Figure 6A). Chromatin immunoprecipitation analysis of p50 and p65 recruitment to the *CXCL1, CXCL2* and *IL8* promoters demonstrated the increased binding of p50 and p65 to these promoters in *NFKB1^S80A^* cells relative to WT cells (Figure 6B–D). Together, these data show that the regulation of p50 binding by S80 is determined primarily by the identity of the −1 nucleotide which appears to modify the binding of both p50:p50 homodimer and p50:p65 heterodimer complexes. Thus S80 phosphorylation reduces the binding of p50 containing NF-κB dimers to κB sites containing an A at the −1 position.

### S80 phosphorylation inhibits transcription from −1A containing κB sites

**Figure 7.**
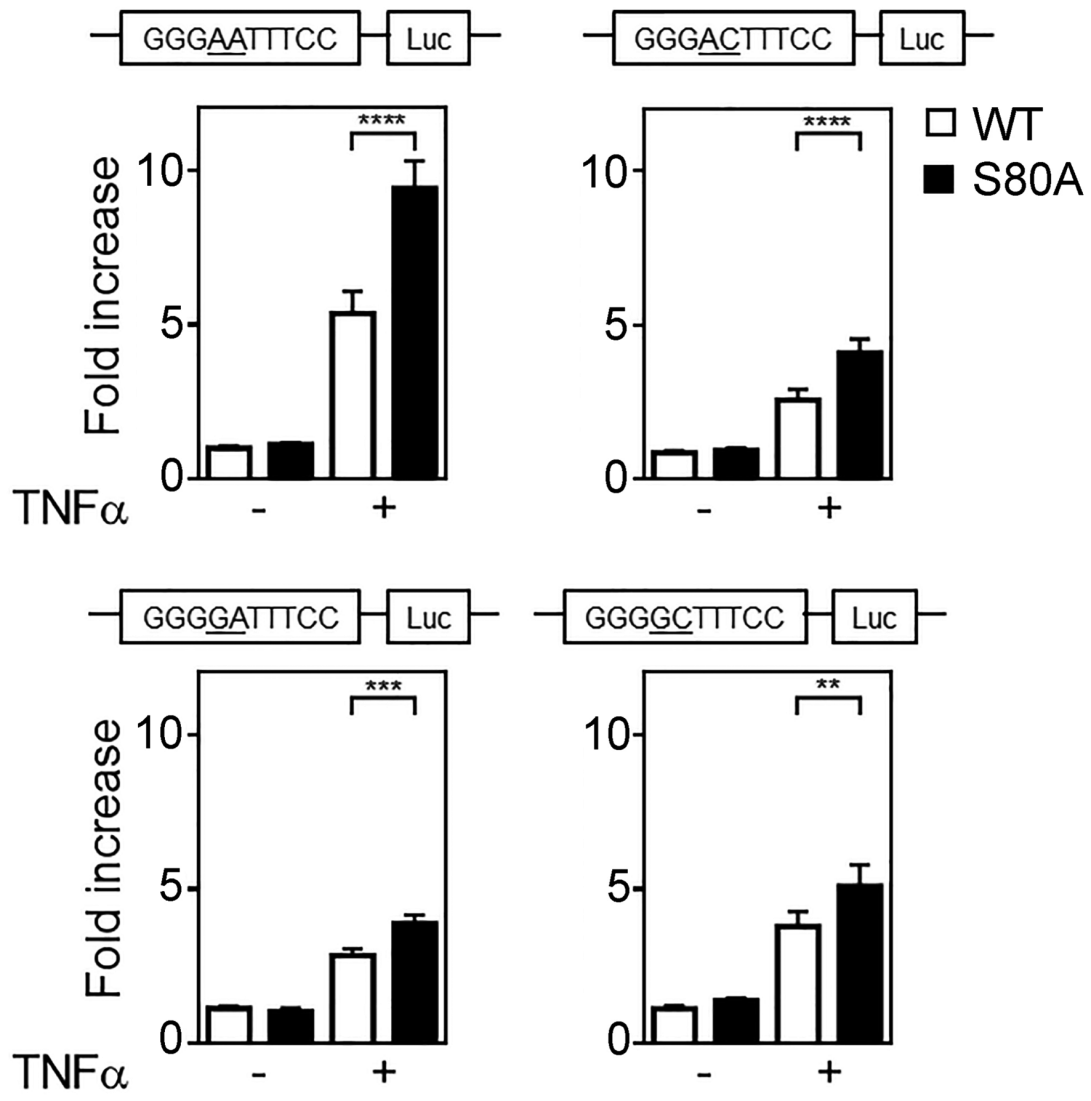
S80 phosphorylation regulates transcription in a κB sequence specific manner. WT and *NFKB1^S80A^* HEK293T cells were transfected with pTAL-NF-κB reporter constructs with four identical tandem κB-sites that vary at the −1 and −2 positions as indicated (underlined). Twenty four hours post transfection, cells were either left untreated or treated with TNFα for 8 h before luciferase activity was measured. The *Renilla* luciferase expression vector pRLTK was used as an internal control to normalize transfection efficiency across all samples. Reporter activity is represented as fold increase over untreated WT cells. Data shown are mean ± S.E. of quadruplicate samples. Statistical significance between treated WT and *NFKB1*^S80A^ cells was determined by Student's *t* test. **P ≤ 0.01; ***P ≤ 0.001; ^****^P ≤ 0.0001.

The transcriptomic and DNA binding analyses suggest that the increased transcription of pro-inflammatory genes in *NFKB1^S80A^* cells results from increased binding of p50^S80A^ containing NF-κB dimers to promoter κB sites containing an A in the −1 position. We next sought to investigate whether increased DNA binding of p50^S80A^ is sufficient to increase target gene transcription or whether the transcriptional outcome observed in TNFα treated *NFKB1^S80A^* cells occurs only in the context of the gene promoter. We generated 4 different luciferase reporter constructs that contained four κB site repeats that vary at either the −2 or the −1 position immediately upstream of a minimal promoter and firefly luciferase reporter gene. The 4 reporter plasmids each contained a κB site sequence identical to each of the sequences employed in the DAPA experiments described above (Figure 6). WT or *NFKB1^S80A^* cells were transiently transfected with the different reporter plasmids along with a constitutive expression vector for *Renilla* luciferase to enable normalisation for transfection efficiency. Luciferase reporter activity was measured in untreated and TNFα-treated cells to determine the impact of p50^S80A^ on transcription driven by specific κB site sequences. TNFα-induced luciferase activity from the reporter containing the GGGA(−2)A(−1)TTTCC sequence was approximately 2 fold greater in *NFKB1^S80A^* cells compared to WT cells (Figure 7), consistent with the increased DNA binding of p50^S80A^ to this sequence observed in the DAPA assays (Figure 6A). Interestingly, TNFα induced reporter activity in both WT and *NFKB1^S80A^* cells is highest in reporter plasmids containing the GGGA(−2)A(−1)TTTCC site compared to the other κB sites tested, suggesting that κB sites containing an A at the −1 and −2 position are more potent drivers of gene transcription. The importance of the −1 nucleotide is further highlighted by the observed overall decrease in luciferase activity in both WT and *NFKB1^S80A^* cells when the −1A is changed to −1C (Figure 7).

The increased TNFα induced reporter activity from the A(−2)C(−1) κB site seen in *NFKB1^S80A^* cells relative to WT cells (Figure 7) is also consistent with the observed increased expression levels in *NFKB1*^S80A^ cells of genes which contain either A(−2)A(−1) or A(−2)C(−1) κB sites (Figure 5A). The relative differences in TNFα induced reporter activity between WT and *NFKB1^S80A^* cells are much less where the κB site contains a G(−2)A(−1) and G(−2)C(−1) sequence (Figure 7). This is consistent with the transcription profiles observed for genes that contain either G(−2)A(−1) or G(−2)C(−1) κB sites where expression is similar between WT and *NFKB1*^S80A^ cells (Figure 5B). Furthermore, DNA binding analysis also showed that p65 binding to these sites is unaffected by S80 phosphorylation which correlates with the reporter activity (Figure 6). The reporter activity for all four κB sequences tested were equivalent between untreated WT and *NFKB1^S80A^*cells, consistent with TNFα dependent activation of IKKβ as a requirement for p50 phosphorylation. Taken together, these data show that S80 phosphorylation regulates NF-κB transcriptional activity in a κB sequence specific manner and that binding site sequence differences are sufficient to determine transcriptional outcome following S80 phosphorylation. Specifically, S80 phosphorylation acts to limit the NF-κB mediated transcription of genes containing a −1A κB site nucleotide.

## DISCUSSION

In this study, we have identified S80 as a novel phosphorylation site on the NF-κB p50 subunit. The phosphorylation of S80 by the IKKβ kinase also identifies p50 as a novel substrate for this kinase. Our data demonstrates that the phosphorylation of p50 at S80 selectively regulates TNFα-induced transcription by regulating the DNA binding of p50 at κB sites in a sequence specific manner. Thus, phosphorylation of S80 reduces p50-DNA binding to κB sites with a −1A, and thereby limits the expression of genes under the control of regulatory elements bearing this sequence. The regulation of p50 DNA binding by S80 phosphorylation occurs both in the context of p50:65 heterodimers and p50:p50 homodimers, revealing the regulation of the transcriptional activity of the p65 subunit *in trans* through the modification of p50.

In addition to the central role of IKKβ as an activator of the NF-κB pathway through the phosphorylation of IκBα, IKKβ also phosphorylates a number of other components of the NF-κB pathway (8). This includes NF-κB p105, which is phosphorylated by IKKβ at the C terminus to promote its limited proteasomal degradation to generate the p50 subunit of NF-κB. NF-κB p65 is also phosphorylated by IKKβ at S468 (34) and S536 (34–38) which serves to regulate p65 transcriptional activity.

Our identification of p50 as an IKKβ substrate places p50 alongside these other components of the NF-κB pathways as a regulatory target of IKKβ kinase activity. Of note, the regulation of sequence specific p50 DNA binding by IKKβ phosphorylation reveals a novel mechanism of IKKβ-mediated control of NF-κB activity. The phosphorylation of p65 at S468 and S536 by a number of other kinases in addition to IKKβ (39) suggests that S80 of p50 is also likely to be phosphorylated by other kinases. IKKβ phosphorylation of S80 would be expected to occur following stimulation of cells by other IKK activating stimuli in addition to TNFα (e.g Toll-like receptors, antigen receptors etc.). However, it is possible that IKKβ-mediated phosphorylation of S80 could be modulated by the phosphorylation of other sites of p50, thereby enabling signal specific control of S80 phosphorylation through pathway-specific activation of other p50 kinases. The future identification of additional S80 kinases will shed further light on the role of S80 phosphorylation in the regulation of NF-κB dependent transcriptional responses in the context of different cellular stimuli.

The NF-κB barcode hypothesis proposes that post-translational modifications of NF-κB subunits, either alone or in combination, generate distinct functional states that direct transcription in a gene specific manner (40). This hypothesis has largely been generated from studies of p65 phosphorylation which indicate that individual sites of p65 phosphorylation may regulate the expression of distinct subsets of NF-κB target genes (40). The molecular basis for such gene specific effects of p65 phosphorylation are not clear in many cases. Although studies have demonstrated that S468 phosphorylation promotes the ubiquitination of p65 by enhancing interaction with an E3 ligase complex containing COMMD1, GCN5, Cullin2 and SOCS1 at certain promoters (41), it is not understood what directs this interaction at these specific promoters. In this study we establish that the gene-specific transcriptional effects of S80 phosphorylation are mediated by the differential binding of phosphorylated p50 with κB sites containing an A nucleotide at the −1 position. Importantly, p50 S80 phosphorylation primarily affects the binding of p50:p65 heterodimers at these sites to inhibit gene transcription. While our data does not identify S80 phosphorylation as a regulator of p50 homodimer function, further analysis will be required to determine whether cell or signal specific factors control dimer specific effects of S80 phosphorylation. Phosphorylation of p50 at S328 has previously been demonstrated to inhibit the binding of p50 to κB sites containing a C nucleotide at the −1 position (14). This study, together with our data, identifies the −1 position of κB sites as a critical factor in determining the transcriptional consequences of p50 phosphorylation. These data also establish κB site sequence as an additional and important component to be considered in further developing the NF-κB barcode hypothesis.

DNA binding sites can act as allosteric regulators of transcriptional regulators (42). Distinct DNA conformations adopted by particular κB sequences provides a potential mechanism to explain why single nucleotide variations affect DNA binding of NF-κB dimers. In support of this an *in silico* transcription factor binding site shape analysis of the κB sites employed in our experiments predicts a unique conformation for each κB site (25) (Supplementary Figure S4). Previous studies have revealed that phosphorylation of p65 induces conformational changes that may influence the transcriptional outcome following DNA binding (43). Using similar approaches we assessed conformational differences between p50^WT^ and p50^S80A^ by limited proteolytic digestion (Supplementary Figure S5). This analysis showed different sensitivities to digestion between p50^WT^ and p50^S80A^, indicating that S80 phosphorylation may induce a conformational change that could modify the binding of p50 dimers to κB sites containing an A nucleotide in the −1 position. Such conformational changes may modify the transactivating potential of NF-κB dimers by modifying DNA binding to specific sites, but also possibly by modifying interaction with other factors that in turn affect DNA binding.

Our data demonstrate that the interaction of S80 phosphorylated p50 with κB sites containing an A at the −1 position is sufficient to inhibit DNA binding and gene transcription of associated genes and does not necessarily require the context of a promoter. Interestingly, our data also show that individual κB sequences have different capacities to drive transcription as measured by reporter assays incorporating κB sequences upstream of a minimal promoter. These analyses identified −2A, −1A κB sites as more potent drivers of transcription than other sites tested. Remarkably, the increased transcription from reporter vectors containing these κB sites is also reflected in TNFα stimulated cells where genes regulated by these sites are induced at significantly higher levels than genes regulated by other κB sites. Indeed, these κB sites appear to be enriched in the regulatory regions of genes encoding pro-inflammatory cytokines and chemokines, including TNFα, IL-8 and the chemokines CXCL1 and CXCL2. The enrichment of specific κB sites in a functional class of genes provides strong evidence that NF-κB phosphorylation and κB sequences may establish regulatory networks to coordinate stimulus-specific transcriptional programmes.

In conclusion, this study describes a novel site of IKKβ phosphorylation of the p50 subunit that regulates TNFα induced transcriptional responses in a gene-selective manner. Our data demonstrates that the gene selective effect of S80 phosphorylation on transcription is encoded in the κB sequence, specifically by the −1 nucleotide position. The results of this study contributes further to our understanding of the regulation of transcriptional programmes by NF-κB. Future research may enable the prediction of transcriptional outcome based on an understanding of the phosphorylation status of NF-κB and DNA binding site sequence.

## DATA AVAILABILITY

Heat maps of gene groups were generated using the online tool Morpheus, available at https://software.broadinstitute.org/morpheus. Web Logos were generated using the online tool Weblogo, available at https://weblogo.berkeley.edu/logo.cgi. Transcription factor binding site shape analysis was performed using the online tool TFBSshape available at available at http://rohslab.cmb.usc.edu/TFBSshape/. RNA-seq data is available in the NCBI Gene Expression Omnibus database with the accession number GSE117279.

## Supplementary Material

gkz873_Supplemental_Files
