# Supplementary material for: The regulation of sequence specific NF-κB DNA binding and transcription by IKKβ phosphorylation of NF-κB p50 at serine 80
Source: Nucleic Acids Res. 2019 Oct 10;47(21):11151–63. doi: 10.1093/nar/gkz873 (PMC6868378; doi:10.1093/nar/gkz873)
Supplement: gkz873_Supplemental_Files [file gkz873_supplemental_files.zip › Supplementary data 24 sept 2019.pdf]

Supplementary Data

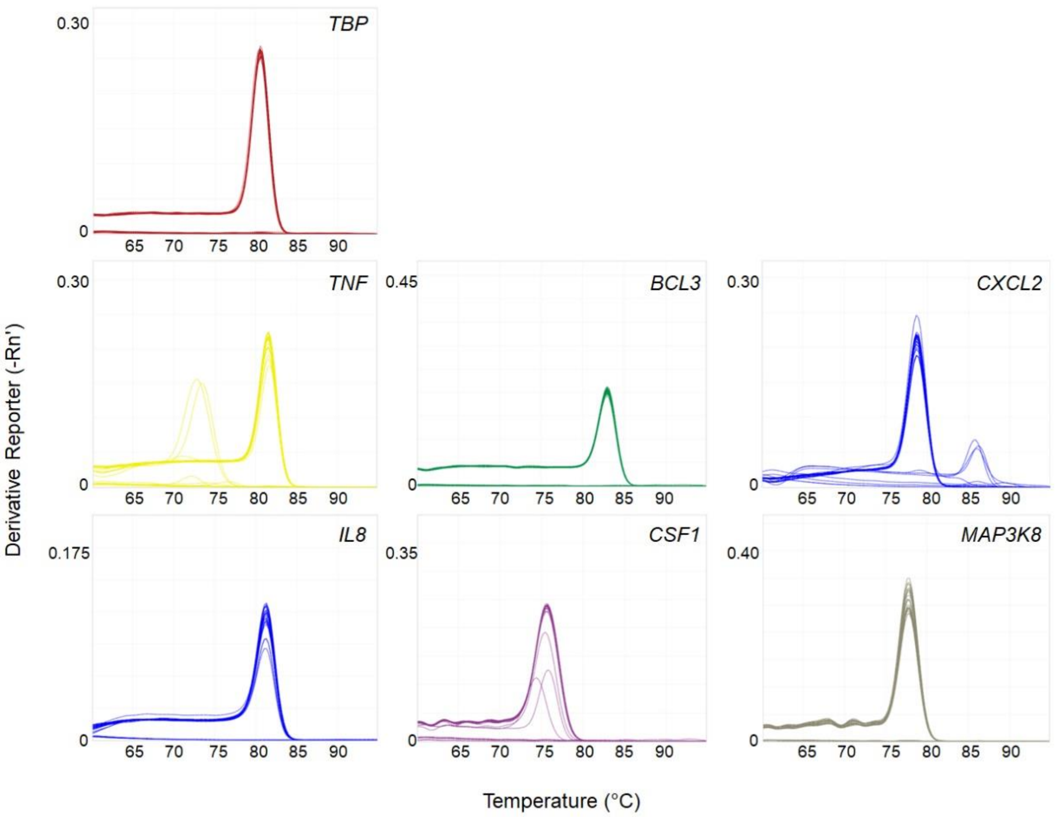

Supplementary Figure S1: Melt curves of qPCR amplicons.
Melt curves of genes analysed by qPCR in this study. Melt curves shown correspond to the representative replicate shown in Figure 4C. Thermocycling conditions for melt curve analysis consisted of 95°C for 15 s, 60°C for 1 min and 95°C for 15 s.

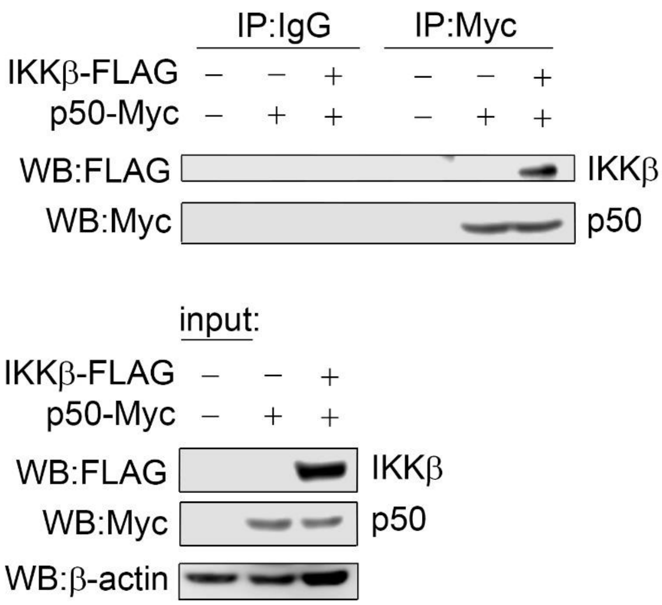

Supplementary Figure S2. Co-immunoprecipitation of p50 and IKKβ. HEK293T cells were co-transfected with Myc-p50 and FLAG-IKKβ as indicated. Equal amounts of lysates were immunoprecipitated with an irrelevant IgG or anti-Myc antibody as indicated and analysed by western blot (WB) using the indicated antibodies.

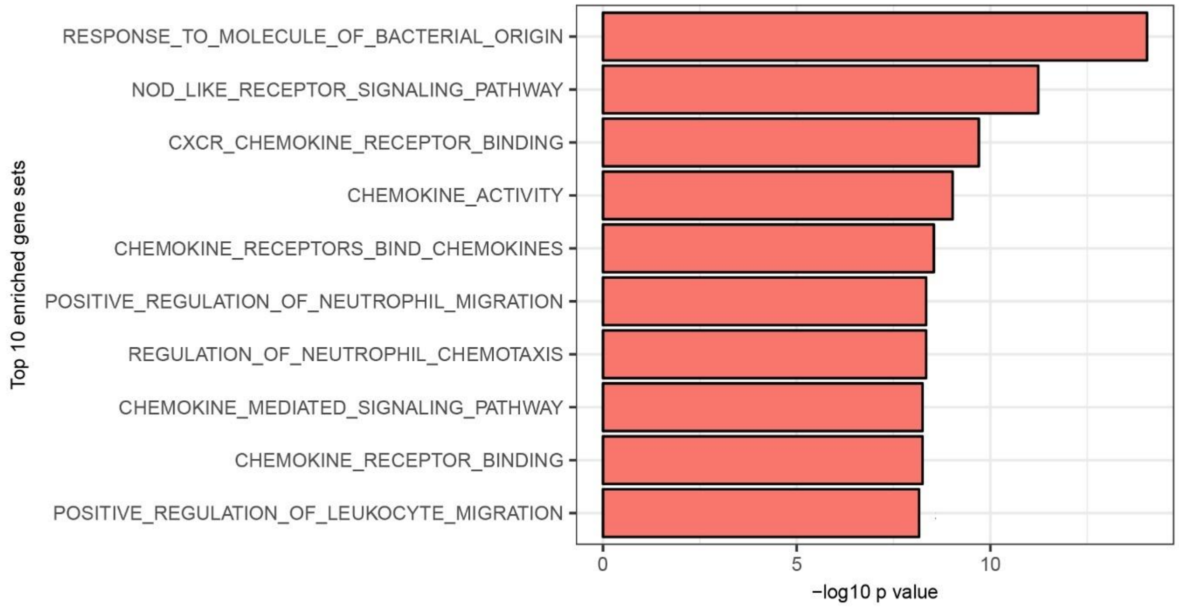

Supplementary Figure S3. Gene ontology of genes differentially expressed in TNFα stimulated S80A cells relative to WT controls. Top 10 enriched genes sets are shown.

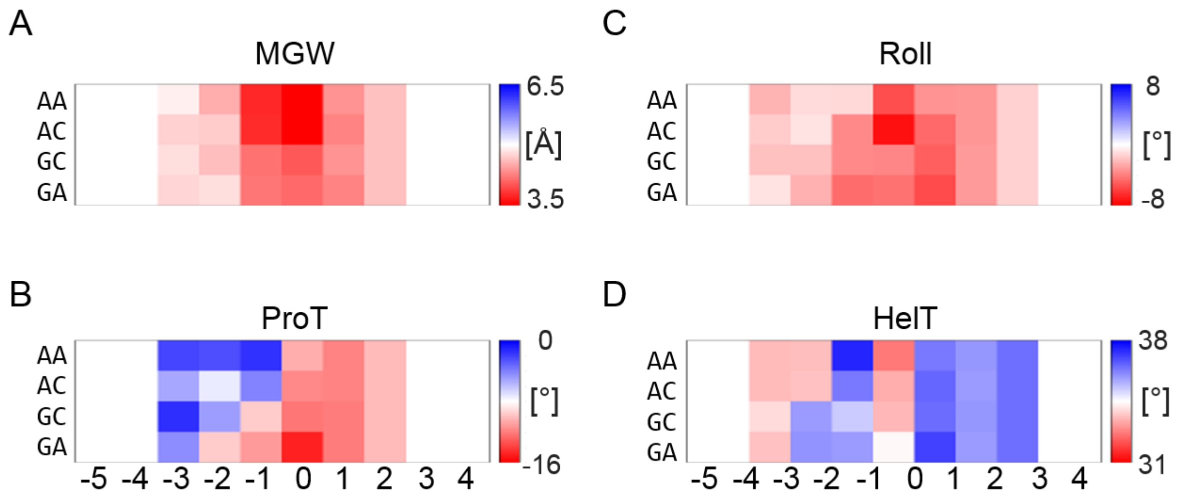

Supplementary Figure S4: Transcription factor binding site shape analysis
Predicted DNA shape values for minor groove width (MGW) (**A**), propeller twist (ProT) (**B**), roll (**C**) and helix twist (HelT) (**D**) are displayed as heat maps. Each κB site analysed has the 10bp format GGG(-2)(-1)TTTCC, with nucleotide variations at the -2 and -1 position (indicated).

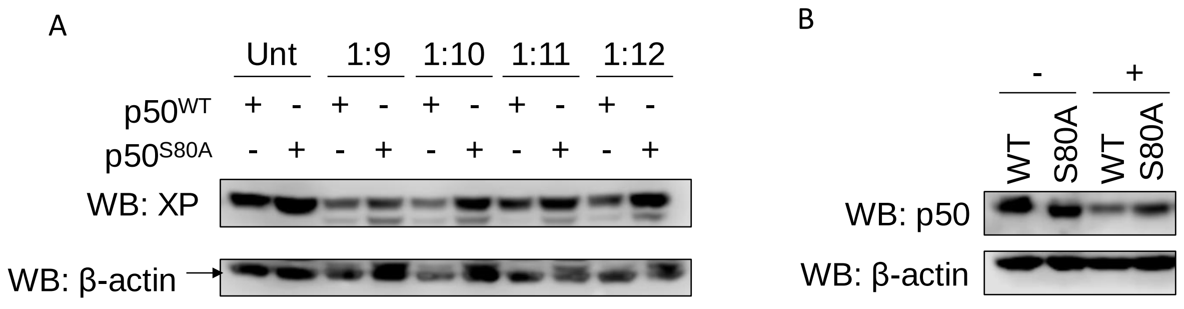

Supplementary Figure 5: S80 affects proteolysis of p50
(**A**) HEK293T cells were transfected with either $p50^{WT}$-XP or $p50^{S78A}$-XP plasmids as indicated. Whole cell lysates were either left untreated (Unt) or treated with varying trypsin:lysate (w/w) ratios as indicated. p50 protein degradation levels were analysed by western blot (WB) using anti-Xpress antibody. (**B**) Whole cell lysates extracted from TNFα treated (10ng/ml) WT or $\mathit{NFKB1}^{S80A}$ cells were left untreated (-) or treated with trypsin at a 1:9 ratio of trypsin:lysate (w/w). p50 protein degradation levels were analysed by western blot (WB) using anti-p50 antibody.
